# Intra-individual comparison of ^68^Ga-PSMA-11 and ^18^F-DCFPyL normal-organ biodistribution

**DOI:** 10.1186/s40644-019-0211-y

**Published:** 2019-05-15

**Authors:** Gonçalo Ferreira, Amir Iravani, Michael S. Hofman, Rodney J. Hicks

**Affiliations:** 1 Nuclear Medicine Department, Instituto Português de Oncologia do Porto, Rua Dr. António Bernardino de Almeida, 4200-072 Porto, Portugal; 2 Centre for Molecular Imaging, Department of Cancer Imaging, Peter MacCallum Cancer Centre, Melbourne, Victoria Australia; 3 Sir Peter MacCallum Department of Oncology, University of Melbourne, Melbourne, Victoria Australia

**Keywords:** ^18^F-DCFPyL, ^68^Ga-PSMA-11, PET/CT, Biodistribution, Prostate cancer

## Abstract

**Purpose:**

Detailed data comparing the biodistribution of PSMA radioligands is still scarce, raising concerns regarding the comparability of different compounds. We investigated differences in normal-organ biodistribution and uptake variability between the two most commonly PSMA tracers in clinical use, ^68^Ga-PSMA-11 and ^18^F-DCFPyL.

**Methods:**

This retrospective analysis included 34 patients with low tumor burden referred for PET/CT imaging with ^68^Ga-PSMA-11 and subsequently ^18^F-DCFPyL. Images were acquired with 4 cross-calibrated PET/CT systems. Volumes of interest were placed on major salivary and lacrimal glands, liver, spleen, duodenum, kidneys, bladder, blood-pool and muscle. Normal-organ biodistribution of both tracers was then quantified as SUV_peak_ and compared using paired tests, linear regression and Bland-Altman analysis. Between-patient variability was also assessed. Clinical and protocol variables were investigated for possible interference.

**Results:**

For both tracers the highest uptake was found in the kidneys and bladder and low background activity was noted across all scans. In the quantitative analysis there was significantly higher uptake of ^68^Ga-PSMA-11 in the kidneys, spleen and major salivary glands (*p* <  0.001), while the liver exhibited slightly higher ^18^F-DCFPyL uptake (*p* = 0.001, mean bias 0.79 ± 1.30). The lowest solid-organ uptake variability was found in the liver (COV 21.9% for ^68^Ga-PSMA-11, 22.5% for ^18^F-DCFPyL). There was a weak correlation between ^18^F-DCFPyL uptake time and liver SUV_peak_ (r = 0.488, *p* = 0.003) and, accordingly, patients scanned at later time-points had a larger mean bias between the two tracers’ liver uptake values (0.05 vs 1.46, *p* = 0.001).

**Conclusion:**

Normal tissue biodistribution patterns of ^68^Ga-PSMA-11 and ^18^F-DCFPyL were similar, despite subtle differences in quantitative values. Liver uptake showed an acceptable intra-patient agreement and low inter-patient variability between the two tracers, allowing its use as a reference organ for thresholding scans in the qualitative comparison of PSMA expression using these different tracers.

**Electronic supplementary material:**

The online version of this article (10.1186/s40644-019-0211-y) contains supplementary material, which is available to authorized users.

## Introduction

Prostate-specific membrane antigen (PSMA) is a transmembrane glycoprotein upregulated in most prostate cancer (PC) cells and its expression levels relate to tumor stage and grade [1–3]. Exploiting this feature, several low-molecular-weight Glu-ureido–based PSMA inhibitor radioligands for Positron Emission Tomography/Computed Tomography (PET/CT) have entered the clinical scope of PC workup [4–6]. These tracers specifically bind to the extracellular domain of PSMA and are internalized, leading to tumor uptake and retention [7, 8].

The most widely used PSMA radiotracer is ^68^Ga-PSMA-11 (also named ^68^Ga-PSMA-HBED-CC) [9], with increasing clinical experience in a variety of PC indications [10–14]. Despite the promise and widespread clinical adoption of this agent, there are logistic issues with use of this tracer related to its short physical half-life (68 min) and decreasing synthesis yields as generators decay. It is also difficult and expensive to comply with good manufacturing practice guidelines and therefore centralized radiopharmacy production and distribution are constrained. These issues have encouraged development of ^18^F-labeled PSMA ligands [15–17], which allow large-scale, cyclotron production and distribution to meet growing demand for molecular imaging evaluation of PC. Among these, the most common agent in clinical use is ^18^F-DCFPyL, which is a second-generation fluorinated PSMA-targeted PET radiotracer [15] already showing promise clinically [18–22].

Besides qualitative assessment of the presence of malignant PSMA-expressing lesions, PSMA PET/CT has an evolving role in the quantitative evaluation of tumor target expression, with potential applications in prognostic stratification, assessment of suitability for PSMA-targeted therapy and subsequent evaluation of treatment response [23–27]. With expected increasing adoption of PSMA-ligand PET/CT into clinical practice and trials, reporting standards are being developed to enhance reproducibility and communication with clinicians [28]. For this purpose, PSMA expression categories could be defined in relation to reference organs including blood pool, liver and parotid glands. Reference organs have also been used for assessment of suitability of patients for PSMA-targeted therapy [29]. However, detailed data on comparison of biodistribution of different PSMA-ligands is scarce and molecular diversity across the range of available radiotracers is expected to impact tissue kinetics [5, 30], raising concern regarding the generalization of observations and comparability of quantitative data.

In this study we explored ^68^Ga-PSMA and ^18^F-DCFPyL biodistribution in a routine clinical setting, making an intra-individual comparison of normal-organ uptake between the two radiotracers. Patients with low tumor burden were selected to minimize the impact of tumor-sink effect on normal tissue biodistribution.

## Materials and methods

### Patients

We retrospectively reviewed 43 consecutive adult male patients who had undergone PET/CT scans with both ^68^Ga-PSMA-11 and ^18^F-DCFPyL at Peter MacCallum Cancer Centre, between October 2014 and April 2018. All scans were performed as part of routine clinical work-up. From this cohort, 34 patients were included in this study according to the following criteria: 1) low tumor burden, defined as a prostate-specific antigen (PSA) < 20 ng/mL; 2) stable disease between scans, defined as an interval PSA change < 10 ng/mL; 3) no appreciable altered biodistribution on imaging, taking into account absence of radiopharmaceutical infiltration, sink effect due to high tumor burden or renal impairment. We also recorded clinical data, namely baseline PC characteristics, PSA level at the time of each scan and previous therapies. This research has been approved by the institutional ethics committee and patient consent was waived (approval number: 15/46R).

### Imaging procedures

In all patients ^68^Ga-PSMA-11 PET/CT was performed first, with a median time interval between scans of 22.5 months (IQR 15.66–27.75). Patients were administered ^68^Ga-PSMA-11 (1.6 ± 0.41 MBq/Kg) or ^18^F-DCFPyL (3.6 ± 0.18 MBq/Kg) by intravenous injection. Most patients (21/34 of ^68^Ga-PSMA-11 and 32/34 of ^18^F-DCFPyL scans) received intravenous iodinated contrast 10–15 min prior to imaging (50 ml Iohexol 37.75 g/50 ml with 100 ml saline) according to our previously published Computed Tomography (CT) urography protocol [31]. No furosemide was used. Imaging was performed using 4 PET/CT systems: Biograph 64 (Siemens Healthcare, Erlangen, Germany), Discovery 690 and 2 Discovery 710 (GE Healthcare, Milwaukee, WI). CT was performed first, with no breath-hold to reduce mismatch with PET images. CT parameters were 120 KeV, maximum 220 mAs with automatic dose modulation, section width of 3.75 mm, 0.5 s/rotation, noise index 25 and standard window reconstruction. PET imaging was performed in 3D mode from mid-thigh to vertex, patient positioned with the arms up. Images were acquired from median 57 (IQR 47–68.75) and 91 (IQR 81.25–123) minutes after injection of ^68^Ga-PSMA-11 or ^18^F-DCFPyL, respectively. Bed times were adjusted to patients’ weight (< 64 Kg: 1.5 min/bed; 65–84 Kg: 2 min/bed; 85–100 Kg: 2.5 min/bed; > 100 Kg: 3.5 min/bed), with 8–10 beds acquired. Image reconstruction encompassed ordered subset expectation maximization (OSEM) iterative reconstruction algorithm and Gaussian filter application. Routine quality assurance phantoms confirmed that PET images from the different scanners were quantitatively comparable and also tested ^68^Ga dose calibration accuracy as part of a multi-center trial [32, 33].

### Image analysis

**Fig. 1:**
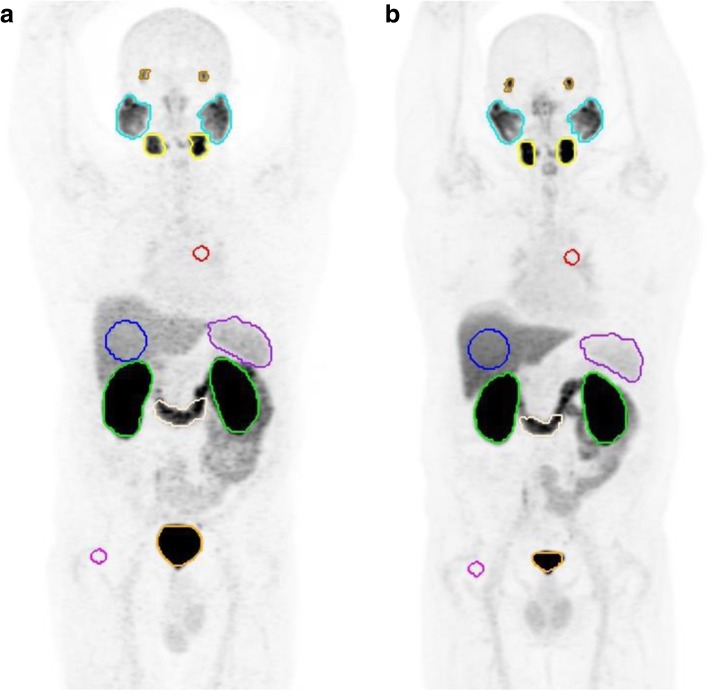
Maximum intensity projection images of both scans ([**a**] 68Ga-PSMA-11; [**b**] 18F-DCFPyL) with representative VOIs in each of the target organs

Image analysis was performed by a Nuclear Medicine physician using an appropriate workstation and vendor neutral software (MIM Encore™ version 6.7, MIM Software Inc., Cleveland, USA). Volumes of interest (VOIs) were automatically drawn over entire organs of moderate to intense physiologic uptake and/or tracer accumulation, namely the major salivary and lacrimal glands, duodenum (third portion), spleen, kidneys (cortex) and urinary bladder, using a gradient-based contouring tool (PET Edge®). Additionally, spherical VOIs were drawn inside the parenchyma of the right hepatic lobe (6 cm diameter), descending thoracic aorta and right gluteus muscle (each 2 cm diameter) – Fig. 1. Tracer biodistribution was then quantified by the peak standardized uptake value (SUV_peak_) as defined in PERCIST criteria [34], which yields less intra-patient bias compared to SUV_max_ and, unlike SUV_mean_, does not require definition of tumor boundaries [35]. For paired organs, the arithmetic mean is presented.

### Statistical analysis

Statistical analysis was performed using SPSS 25.0 (IBM Corp., Armonk, NY) and GraphPad Prism 7 (GraphPad Software, La Jolla, USA). Normality was tested using the Shapiro-Wilk test. Intra-patient comparison of quantitative uptake between the two tracers in each of the target organs encompassed Paired *t* or Wilcoxon signed-rank tests, linear regression and Bland-Altman analysis. Inter-patient variability was assessed by taking the coefficient of variation (COV). Interference of clinical and protocol variables was also investigated, using Spearman’s rank correlation, and by subgroup analysis comparing the quantification biases in a group of patients where imaging with the two tracers was done within a similar time-frame and another with higher ^18^F-DCFPyL uptake duration (Independent samples *t* or Mann-Whitney U test). To account for multiple tests, the significance level used was *p* ≤ 0.01 (two-tailed).

## Results

Patient characteristics are summarized in Table 1. Visual assessment of the expected biodistribution of both tracers was considered normal for all but one patient, who had a past history of right nephrectomy prior to both scans. There was no evidence of malignant involvement within the target organs. Most patients (67.6%) had low volume malignant PSMA expressing lesions on either scan (prostatic/prostate bed in 20.6%, nodal in 44.1% and bony in 17.6% of patients). The remaining 11 (32.4%) patients had no evidence of disease on both exams. The median absolute difference in PSA levels at the time of each scan was 2.75 (IQR 0.43–3.18) ng/mL.

**Table 1:** Patient Characteristics

Variable	N (%) or Median (IQR)
Age (years)	67.5 (9.75)
Baseline risk (NCCN)	
Low	3 (8.8%)
Intermediate	7 (20.6%)
High	21 (61.8%)
Unknown	3 (8.8%)
Indication (at first scan)	
Diagnosis / Primary Staging	2 (5.9%)
Biochemical Recurrence	27 (79.4%)
Restaging of Metastatic Disease	5 (14.7%)
Treatment Naive	2 (5.9%)
Primary Therapy	32 (94.1%)
Radical Prostatectomy	22 (64.7%)
Primary EBRT	5 (14.7%)
Brachitherapy	5 (14.7%)
Previous Salvage Therapy	14 (41.2%)
Salvage EBRT	13 (38.2%)
Salvage LND	1 (2.9%)
Previous Systemic Therapy	13 (38.2%)
ADT	13 (38.2%)
Chemotherapy	1 (2.9%)
Interval Therapy^a^	9 (26.5%)
Local	6 (17.6%)
Systemic	3 (8.8%)
Time between scans (months)	22.5 (12.08)
On ADT at ^68^Ga-PSMA-11 scan	8 (23.5%)
On ADT at ^18^F-DCFPyL scan	10 (29.4%)
PSA at ^68^Ga-PSMA-11 scan (ng/mL)	1.9 (4.44)
PSA at ^18^F-DCFPyL scan (ng/mL)	2.0 (3.55)

^a^Four patients had interval salvage EBRT and 2 had LND; Two patients interval started ADT and 1 patient had interval chemotherapy also starting ADT; one patient interval stopped ADT

*NCCN* National Comprehensive Cancer Network, *EBRT* External Beam Radiotherapy, *LND* Lymph Node Dissection, *ADT* Androgen Deprivation Therapy, *PSA* Prostate Specific Antigen

### Organ uptake comparison

**Fig. 2:**
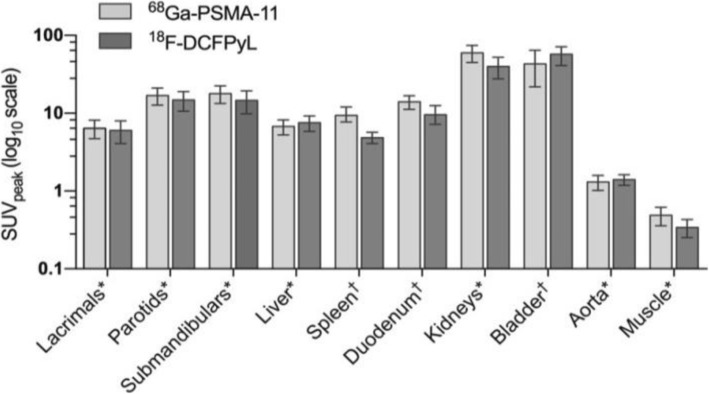
Clustered bar chart of normal-organ SUVpeak with either tracer (68Ga-PSMA-11 and 18F-DCFPyL). For data normally distributed: *mean with stardard deviation error bars; for data not normally distributed: †median with interquartile range error bars. Plotted on a logarithmic scale (log10)

Normal-organ biodistribution was grossly equivalent for both tracers. The highest activities were observed in the kidneys and bladder, followed by the salivary glands. Liver, spleen and proximal small bowel also showed prominent uptake using both tracers and low background activity was noted in the blood-pool (thoracic aorta) and muscle across all scans. These observations conformed with the quantitative uptake values (SUV_peak_) for each tracer (Fig. 2).

**Fig. 3:**
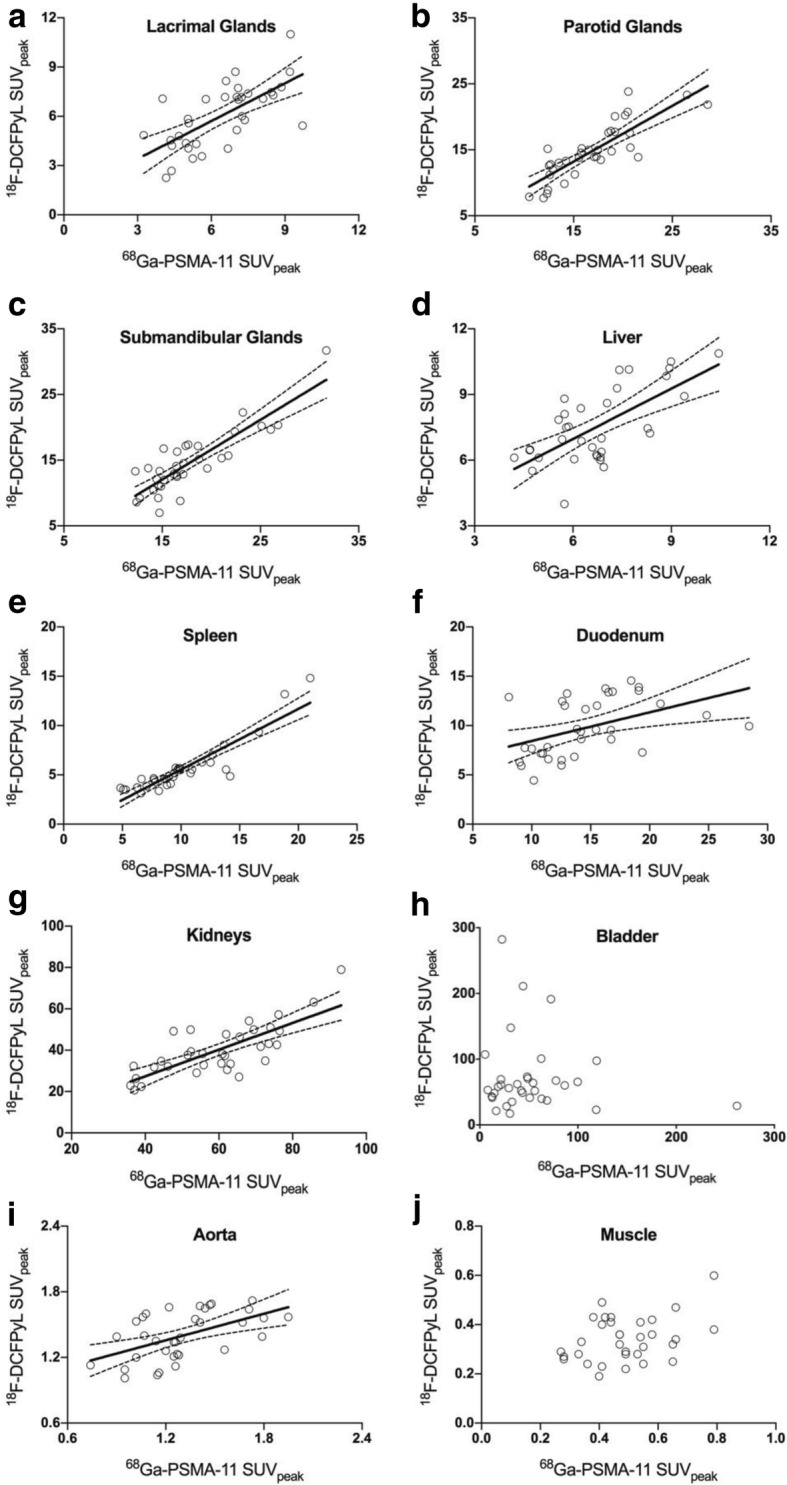
Scatter Plotts depicting the relation of quantitative uptake values (SUVpeak) between the two scans in each of the target organs (y axis: 18F-DCFPyL SUVpeak; x axis: 68Ga-PSMA-11-SUVpeak). Statistically significant correlations (*p* < 0.01) show the corresponding regression lines and 95% CI for the slope

**Fig. 4:**
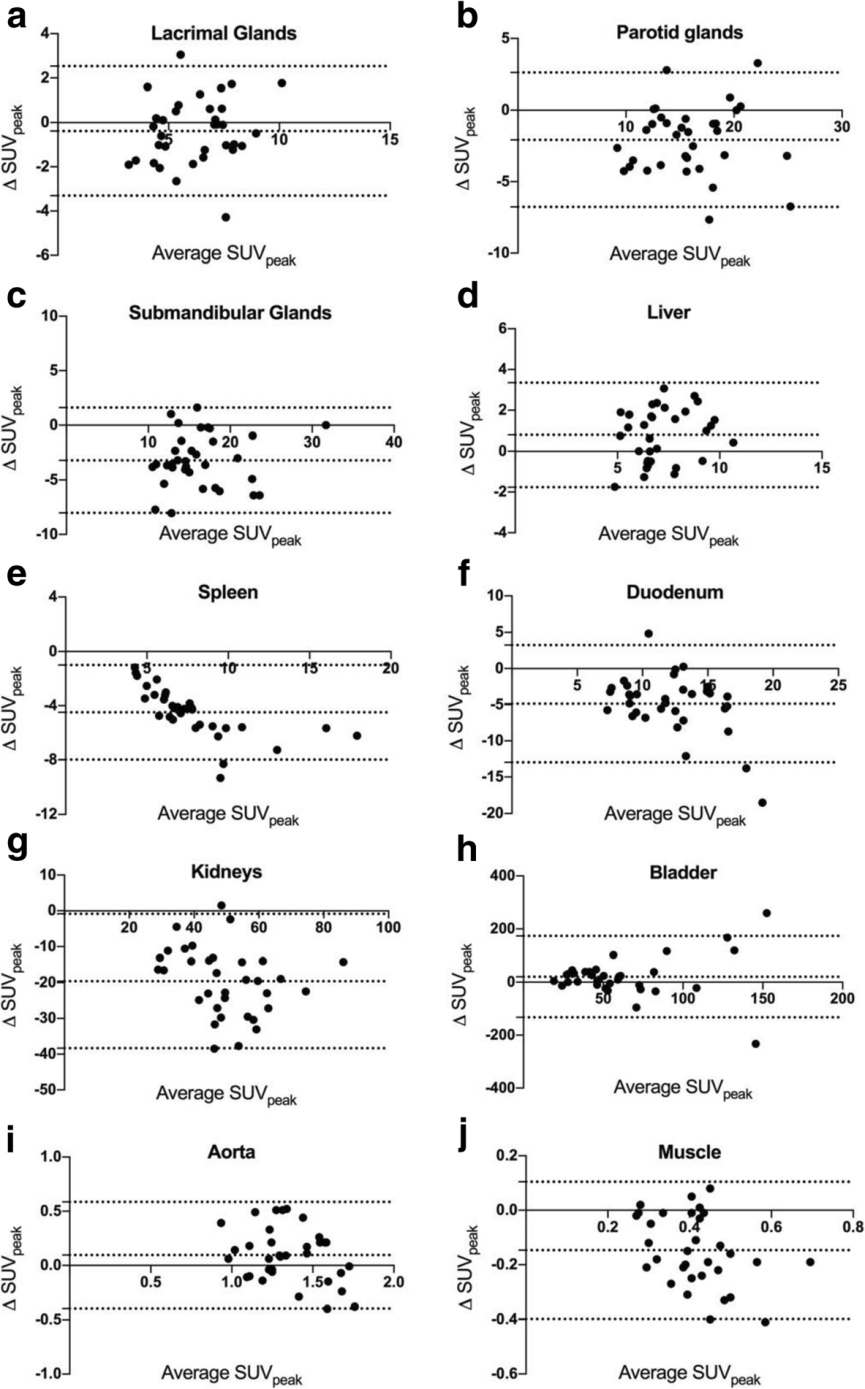
Bland-Altman Plotts showing the differences between quantitative uptake values (ΔSUVpeak, determined by 18F-DCFPyL SUVpeak – [minus] 68Ga-PSMA-11-SUVpeak) between the two scans (y axis) against their average - Average SUVpeak (x axis). Plotted dotted lines represent the mean Bias in the entire cohort and 95% limits of agreement

Despite the visually appreciable similarities, there were subtle but statistically significant differences in the quantitative analysis of normal-organ uptake amidst the two agents. Detailed comparison is described in Table 2 and depicted in Figs. 3 and 4. Liver tracer quantification between tracers was well correlated, allowing for some variability on a per-patient basis (Fig. 3d). Quantitative uptake was slightly higher in ^18^F-DCFPyL scans (mean SUV_peak_ 7.5 vs 6.7, *p* = 0.001), associating with a mean bias of 0.79 ± 1.30 (Fig. 4d). In contrast, the spleen presented significantly higher ^68^Ga-PSMA-11 uptake values (median SUV_peak_ 9.4 vs 4.9, *p* <  0.001). While splenic activity quantification also correlated well (Fig. 3e), there was an increasing proportional bias at higher uptake values (Fig. 4e, r = 0.681, p <  0.001, mean bias − 4.49 ± 1.78). Tracer activity in the renal cortex was also significantly higher in ^68^Ga-PSMA-11 scans (mean SUV_peak_ 59.6 vs 40.0, p <  0.001), with a mean bias of − 19.60 ± 9.52 (Fig. 4g). Lacrimal and major salivary glands had a good correlation in quantitative uptake between the two scans (Fig. 3a, b, c) and an acceptable overall agreement with a calculated mean bias of − 0.39 ± 1.49 in lacrimal, − 2.08 ± 2.39 in parotid, and − 3.21 ± 2.46 in submandibular glands (Fig. 4a, b, c).

**Table 2:** Comparison of ^68^Ga-PSMA-11 and ^18^F-DCFPyL quantitative uptake in each of the target organs

Target Organ	^68^Ga-PSMA-11 SUV_peak_		^18^F-DCFPyL SUV_peak_		Paired test *P* value	Linear Regression			Bland-Altman	
	Mean (SD) or Median (IQR)	CoV (%)	Mean (SD) or Median (IQR)	CoV (%)		Slope	R^2^	P value	Mean Bias	SD
Lacrimal Glands	6.4 (1.71)^a^	26.7	6.0 (1.95)^a^	32.3	0.140^b^	0.767	0.456	**< 0.001**	−0.39	1.489
Parotid Glands	16.9 (4.16)^a^	24.6	14.8 (4.20)^a^	28.4	**< 0.001** ^b^	0.845	0.699	**< 0.001**	−2.08	2.394
Submandibular Glands	17.9 (4.53)^a^	25.4	14.6 (4.78)^a^	32.7	**< 0.001** ^b^	0.910	0.743	**< 0.001**	−3.21	2.458
Liver	6.7 (1.48)^a^	21.9	7.5 (1.69)^a^	22.5	**0.001** ^b^	0.767	0.449	**< 0.001**	0.79	1.305
Spleen	9.4 (3.84)^c^	36.8	4.9 (1.53)^c^	45.3	**< 0.001** ^d^	0.616	0.820	**< 0.001**	−4.49	1.781
Duodenum	14.0 (5.37)^c^	30.9	9.6 (5.18)^c^	29.8	**< 0.001** ^d^	0.291	0.203	**0.008**	−4.87	4.138
Kidneys	59.6 (14.77)^a^	24.8	40.0 (12.43)^a^	31.1	**< 0.001** ^b^	0.645	0.587	**< 0.001**	−19.60	9.561
Bladder	43.1 (41.02)^c^	91.4	57.3 (28.94)^c^	78.9	0.033^d^	−0.128	0.011	0.549	20.36	78.100
Aorta	1.3 (0.29)^a^	21.9	1.4 (0.21)^a^	15.5	0.033^b^	0.404	0.286	**0.001**	0.10	0.250
Muscle	0.5 (0.13)^a^	28.3	0.3 (0.09)^a^	26.7	**< 0.001** ^b^	0.251	0.140	0.029	−0.15	0.128

For data normally distributed: ^a^ mean (SD); ^b^ Paired *t* test. For data not normally distributed: ^c^ Median (IQR); ^d^ Wilcoxon signed-rank test.

*P* values in bold reflect statistical significance

*SD* Standard Deviation, *IQR* Interquartile Range, *CoV* Coefficient of Variation

Within the solid organs, the lowest uptake variability between patients was found in the liver (COV 21.9% for ^68^Ga-PSMA-11 and 22.5% for ^18^F-DCFPyL).

### Variables influencing organ uptake

**Fig. 5:**
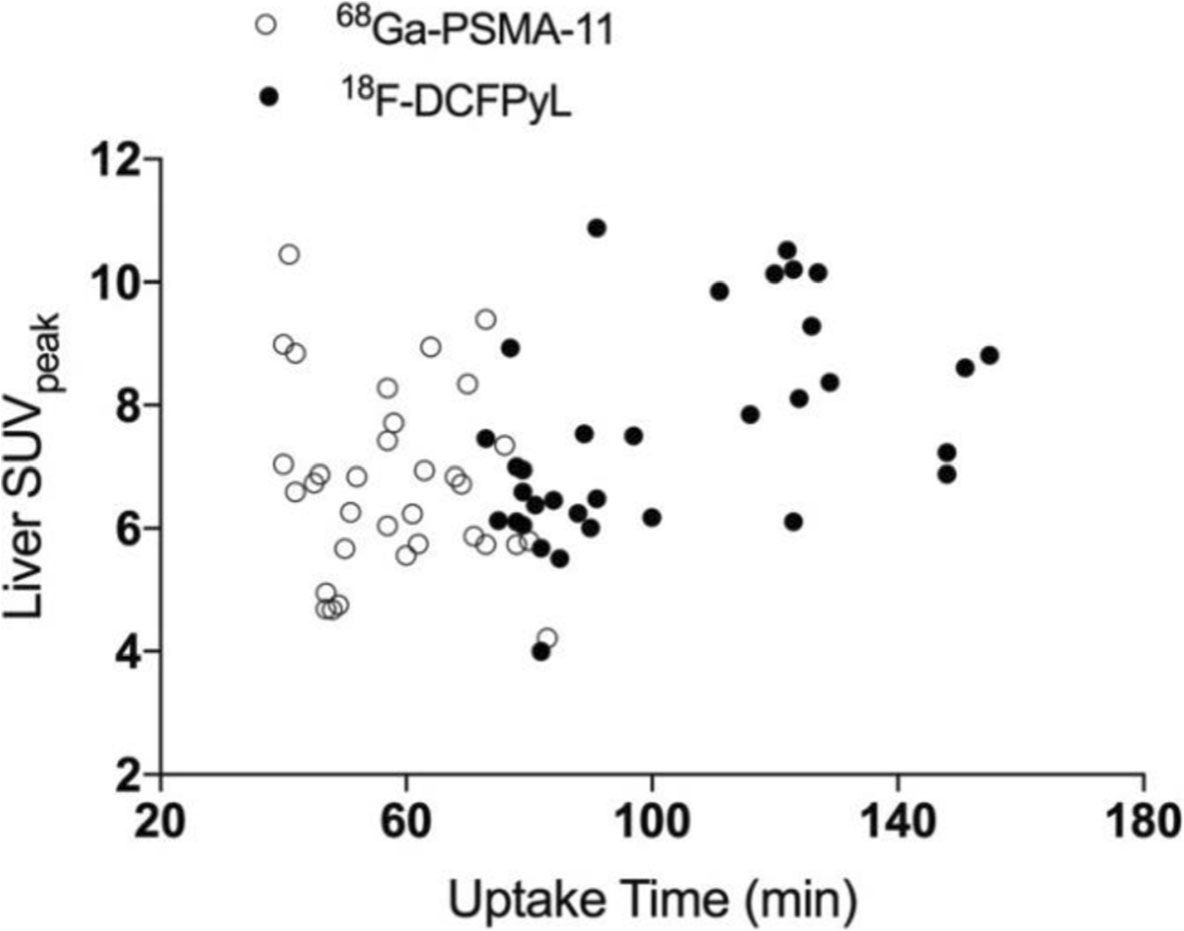
Scatter Plot showing the liver SUVpeak (y axis) plotted against the uptake time (x axis) in each of the two scans

Age, weight, tracer dose (MBq/Kg), uptake time and PSA level were tested for possible correlation with each tracer’s normal-organ uptake (Additional file 1: Table S1). In ^18^F-DCFPyL scans, there was a weak correlation (*r* = 0.488, *p* = 0.003) between uptake time and liver uptake values (Fig. 5) and lacrimal glands SUV_peak_ (r = 0.554, *p* = 0.001). None of the tested variables correlated with ^68^Ga-PSMA-11 uptake within the target organs.

Agreement of uptake quantification between the two tracers was further investigated, taking into account ^18^F-DCFPyL uptake time variability by conducting subgroup analysis (Additional file 1: Table S2). The group of patients scanned with both tracers within a similar time-frame (up to 90 min after injection, *n* = 16) had a statistically significant lower mean bias between the two tracers in liver uptake quantification (0.05 ± 1.122 vs 1.46 ± 1.093, p = 0.001). Furthermore, in both these groups liver COVs of ^18^F-DCFPyL scans were distinctly lower compared to those of the entire study population (16.5 and 18.4% vs. 22.5%), while in ^68^Ga-PSMA-11 scans liver uptake COVs were still similar (20.4 and 22.6% vs. 21.9%).

## Discussion

In this intra-individual comparison of patients scanned with ^68^Ga-PSMA and ^18^F-DCFPyL, the overall biodistribution in normal organs was similar, with both tracers showing specific retention in the salivary and lacrimal glands, small intestine, liver, spleen, kidneys and bladder. While this was somewhat expected, subtle but statistically significant differences were found regarding normal-organ uptake.

The highest uptake was observed in the urinary system in keeping with a predominantly renal clearance for both tracers [9, 19, 36]. Kidney tracer retention was, however, higher in ^68^Ga-PSMA-11 scans, which could relate to specific cortical binding and slower renal clearance for this compound [37]. On the other hand, bladder SUV was higher in ^18^F-DCFPyL scans. This comparison is particularly relevant, as local relapses are common and a diagnostic challenge in the work-up of biochemical recurrence. While slower clearance of ^68^Ga-PSMA-11 could be a possible explanation for this finding, variable hydration, voiding status and non-uniform use of iodinated contrast are important confounders that render interpretation difficult, which is reflected in the very high bladder COV for both scans. Despite this potential limitation, the longer physical half-life of ^18^F allows for delayed imaging with the opportunity for dilution of urinary activity and delayed post-void imaging.

Salivary and lacrimal glands also showed intense uptake and good correlation between the tracers, which increases confidence in inter-scan comparability. Additionally, it was noted that lacrimal gland uptake, unlike salivary gland uptake, seemed to be dependent on ^18^F-DCFPyL uptake time. This is congruent with the hypothesis that later time-point acquisitions may increase the detectability of small structures, which has recently been demonstrated for PC lesions using both ^18^F-DCFPyL [38] and ^18^F-PSMA-1007 [17, 39]. Blood-pool and muscle background activity was very low, providing excellent image quality with both tracers.

To simplify routine clinical practice and mitigate absolute quantification reliability issues between different systems, PSMA expression in a site of local or metastatic disease can be defined according to intensity in relation to the uptake in normal organs. This alternative strategy for PET quantification has been successfully applied in neuroendocrine tumors, with the *Krenning* score defining somatostatin receptor expression [40], and in Lymphoma response assessment using the 5-point scale Deauville criteria [41]. Our group has also described a scoring system for ^18^F-Fluorthymidine [42]. Recently, a similarly pragmatic approach was used in a phase-II study evaluating the efficacy of ^177^Lu-PSMA-617 in men with metastatic castration-resistant PC [29], in which patients were deemed suitable for therapy when lesional ^68^Ga-PSMA-11 uptake was significantly greater than normal liver. Therefore, it is particularly important to access liver uptake agreement between different PSMA tracers. This study demonstrated an acceptable quantitative liver uptake agreement between ^18^F-DCFPyL and ^68^Ga-PSMA-11. However, a weak positive correlation between liver uptake values and ^18^F-DCFPyL uptake time was found and, accordingly, in patients where ^18^F-DCFPyL scans were performed at later time-points there was a significantly larger bias, in which liver uptake was higher than the corresponding scans using ^68^Ga-PSMA-11. Although the optimal time point for ^18^F-DCFPyL imaging is still not fully established, this possible difference might be a consequence of non-plateaued tracer kinetics at the scanning interval and needs to be acknowledged when imaging is performed at later time points.

Moreover, the intrinsic variability of the tracers in normal organs must be well understood in order to be able to attribute tumor signal alterations to real changes in tumor mass, disease progression or response to treatment. The liver was the solid organ with the lowest COVs for both tracers, validating it as an appropriate reference tissue for thresholding images when assessing serial scans. Appropriate image thresholding is vital for qualitative assessment of PET scans [43]. We found a wider range of uptake values within the liver for ^18^F-DCFPyL scans than those observed in another series of the literature where the SUV_mean_ COV was 13.8% [36], but still acceptable in relation to ^18^F-FDG (SUL_mean_ COV 21.0–23.1%) [44]. This probably reflects the wider range of ^18^F-DCFPyL uptake time in this series and a lower population number and may have contributed in some extent to the observed differences between ^68^Ga-PSMA and ^18^F-DCFPyL.

There were some limitations to this study. The cohort was relatively small, included patients undergoing imaging for different indications and there was a non-negligible period between ^68^Ga-PSMA-11 and ^18^F-DCFPyL scans. We minimized possible systematic errors by applying stringent (albeit arbitrary) inclusion criteria. As PSMA-avid tumor burden significantly correlates to PSA levels [24, 45], we excluded patients with high PSA at either scan or significant interval biochemical progression, thus ensuring normal-organ comparability. Furthermore, even with rigorous calibration, the use of 4 different PET/CT systems may influence data output. The retrospective design also impaired optimal protocol coincidence using the two tracers. The mean injected dose and uptake time after tracer injection were higher for ^18^F-DCFPyL, but this variation was considered in the applied statistical analysis and also reflects routine clinical practice since, generally, lower doses and narrower acquisition periods are used for ^68^Ga-labeled tracers. While the optimal time point for ^18^F-DCFPyL is not yet fully established, preliminary data support imaging at 120 min post injection [38]. Accordingly, our ^18^F-DCFPyL protocol has since evolved to imaging at a later time-point, resulting in a somewhat heterogeneous acquisition time-frame for this population. We acknowledged this issue and performed subgroup analysis of physiologic uptake at different time-points. Finally, although there is published data on the change in PSMA expression in tumor lesions following androgen deprivation therapy [46, 47], the effect on normal organ distribution is still unclear. Noticeably, only 3 (8.8%) patients had a systemic treatment commenced and one (2.9%) patient had it stopped in between scans.

This study is not intended to provide a comparison of lesion sensitivity, but there is preliminary, intra-patient comparison data [18, 20]. Head-to-head comparisons between these agents are required to define their relative diagnostic performance but these data further encourage evaluation of ^18^F-DCFPyL given the advantages it provides in terms of radiopharmaceutical supply.

## Conclusion

Normal tissue biodistribution patterns of ^68^Ga-PSMA-11 and ^18^F-DCFPyL were similar, despite subtle differences in quantitative values. Liver uptake demonstrated an acceptable agreement and low inter-patient variability between the two tracers, allowing its use as a reference organ for thresholding scans for qualitative comparison of PSMA expression when using these different tracers.

## Additional file

Additional file 1: **Table S1.** Correlation between clinical/protocol variables and each tracers’ quantitative uptake in the target organs. *P* values in bold reflect statistical significance. **Table S2.** Subgroup comparison of 68Ga-PSMA-11 and 18F-DCFPyL quantitative uptake in each of the target organs taking into account 18F-DCFPyL uptake time variability. (DOCX 31 kb)

## Abbreviations

ADT: Androgen deprivation therapy
COV: Coefficient of variation
CT: Computed Tomography
EBRT: External beam radiotherapy
IQR: Interquartile range
LND: Lymph node dissection
NCCN: National Comprehensive Cancer Network
PC: Prostate cancer
PET: Positron Emission Tomography
PET/CT: Positron Emission Tomography/Computed Tomography
PSA: Prostate-specific antigen
PSMA: Prostate-specific membrane antigen
SD: Standard deviation
SUL: Standardized uptake value normalized to lean body mass
SUV: Standardized uptake value
VOIs: Volumes of interest
