# Supplementary material for: Intra-individual comparison of 68Ga-PSMA-11 and 18F-DCFPyL normal-organ biodistribution
Source: Cancer Imaging. 2019 May 15;19:23. doi: 10.1186/s40644-019-0211-y (PMC6521415; doi:10.1186/s40644-019-0211-y)
Supplement: Supplementary file 1 — Table S1. Correlation between clinical/protocol variables and each tracers’ quantitative uptake in the target organs. P values in bold reflect statistical significance. Table S2. Subgroup comparison of 68Ga-PSMA-11 and 18F-DCFPyL quantitative uptake in each of the target organs taking into account 18F-DCFPyL uptake time variability. (DOCX 31 kb) [file 40644_2019_211_MOESM1_ESM.docx]

**ADDITIONAL FILES DATA**

**ADDITIONAL TABLE 1 –** Correlation between clinical/protocol variables and each tracers’ quantitative uptake in the target organs.

| Target Organ | Scan | Age | | Dose (MBq/Kg) | | Weight | | Uptake Time | | PSA level | |
| --- | --- | --- | --- | --- | --- | --- | --- | --- | --- | --- | --- |
|  |  | r* | P* | r* | P* | r* | P* | r* | P* | r* | P* |
| Lacrimal Glands | *^68^Ga-PSMA-11* | 0.103 | 0.560 | 0.142 | 0.424 | 0.147 | 0.406 | 0.146 | 0.409 | -0.003 | 0.988 |
|  | *^18^F-DCFPyL* | -0.131 | 0.460 | -0.290 | 0.096 | 0.255 | 0.145 | **0.554** | **0.001** | 0.085 | 0.631 |
| Parotid Glands | *^68^Ga-PSMA-11* | 0.123 | 0.487 | -0.060 | 0.738 | -0.036 | 0.841 | -0.044 | 0.807 | 0.106 | 0.551 |
|  | *18F-DCFPyL* | 0.092 | 0.604 | 0.116 | 0.513 | -0.140 | 0.431 | 0.043 | 0.809 | 0.153 | 0.389 |
| Submandibular Glands | *^68^Ga-PSMA-11* | -0.211 | 0.231 | 0.108 | 0.544 | 0.260 | 0.137 | -0.011 | 0.951 | 0.070 | 0.695 |
|  | *^18^F-DCFPyL* | -0.041 | 0.816 | -0.166 | 0.348 | 0.266 | 0.129 | 0.163 | 0.358 | 0.287 | 0.100 |
| Liver | *^68^Ga-PSMA-11* | 0.023 | 0.898 | 0.235 | 0.181 | 0.036 | 0.838 | -0.175 | 0.321 | 0.035 | 0.846 |
|  | *^18^F-DCFPyL* | -0.050 | 0.778 | 0.041 | 0.817 | 0.158 | 0.373 | **0.488** | **0.003** | 0.093 | 0.602 |
| Spleen | *^68^Ga-PSMA-11* | 0.125 | 0.483 | 0.112 | 0.528 | -0.168 | 0.342 | -0.201 | 0.254 | 0.177 | 0.316 |
|  | *^18^F-DCFPyL* | 0.310 | 0.075 | 0.132 | 0.455 | -0.181 | 0.306 | 0.057 | 0.750 | 0.366 | 0.034 |
| Duodenum | *^68^Ga-PSMA-11* | -0.063 | 0.724 | 0.031 | 0.860 | -0.049 | 0.784 | -0.107 | 0.547 | -0.167 | 0.345 |
|  | *^18^F-DCFPyL* | -0.231 | 0.189 | 0.141 | 0.427 | 0.021 | 0.906 | 0.412 | 0.016 | 0.058 | 0.745 |
| Kidneys | *^68^Ga-PSMA-11* | -0.348 | 0.044 | 0.018 | 0.921 | 0.079 | 0.656 | -0.079 | 0.657 | -0.017 | 0.922 |
|  | *^18^F-DCFPyL* | -0.099 | 0.578 | -0.089 | 0.617 | -0.103 | 0.564 | 0.068 | 0.702 | -0.056 | 0.751 |
| Bladder | *^68^Ga-PSMA-11* | -0.039 | 0.827 | -0.200 | 0.257 | 0.233 | 0.184 | 0.244 | 0.164 | 0.097 | 0.584 |
|  | *^18^F-DCFPyL* | 0.037 | 0.837 | -0.058 | 0.744 | 0.288 | 0.098 | -0.029 | 0.871 | -0.074 | 0.679 |
| Aorta | *^68^Ga-PSMA-11* | -0.186 | 0.291 | -0.160 | 0.367 | 0.275 | 0.116 | -0.228 | 0.196 | -0.087 | 0.627 |
|  | *^18^F-DCFPyL* | 0.153 | 0.386 | -0.077 | 0.665 | 0.276 | 0.115 | -0.167 | 0.346 | -0.206 | 0.243 |
| Muscle | *^68^Ga-PSMA-11* | 0.152 | 0.391 | -0.271 | 0.122 | -0.137 | 0.441 | **-0.444** | **0.009** | -0.115 | 0.519 |
|  | *^18^F-DCFPyL* | **0.466** | **0.005** | -0.017 | 0.923 | 0.019 | 0.916 | -0.269 | 0.124 | 0.080 | 0.652 |
| *Spearman correlation test | | | | | | | | | | | |

**ADDITIONAL TABLE 2 –** Subgroup comparison of ^68^Ga-PSMA-11 and ^18^F-DCFPyL quantitative uptake in each of the target organs taking into account ^18^F-DCFPyL uptake time variability.

| Target Organ | ^18^F-DCFPyL Uptake Time  ≤ 90 min (N=16) | | ^18^F-DCFPyL Uptake Time  > 90 min (N=18) | | Independent samples test P value |
| --- | --- | --- | --- | --- | --- |
|  | Mean or Median SUV_peaK_ Bias | SD or IQR | Mean or Median SUV_peaK_ Bias | SD or IQR |  |
| Lacrimal Glands | -1.07* | 1.415* | 0.28* | 1.319* | **0.008^¶^** |
| Parotid Glands | -2.27* | 2.858* | -1.77* | 1.947* | 0.558^¶^ |
| Submandibular Glands | -3.82* | 2.118* | -2.67* | 2.665* | 0.170^¶^ |
| Liver | 0.05* | 1.122* | 1.46* | 1.093* | **0.001^¶^** |
| Spleen | -4.46* | 2.044* | -4.51* | 1.571* | 0.934^¶^ |
| Duodenum | -4.80^†^ | 3.498^†^ | -3.55^†^ | 3.175^†^ | 0.112^ǂ^ |
| Kidneys | -20.48* | 8.511* | -18.82* | 10.590* | 0.616^¶^ |
| Bladder | 9.74^†^ | 64.860^†^ | 24.14^†^ | 31.948^†^ | 0.512^ǂ^ |
| Aorta | 0.17* | 0.295* | 0.03* | 0.185* | 0.098^¶^ |
| Muscle | -0.14* | 0.139* | -0.15* | 0.122* | 0.880^¶^ |
| For data normally distributed: *mean and SD; ^¶^ Independent samples *t* test. For data not normally distributed: ^†^ Median and IQR; ^ǂ^ Mann-Whitney U test. | | | | | |
| SD – Standard Deviation; IQR – Interquartile Range | | | | | |
